# Single-Cell RNA Sequencing of Metastatic Testicular Seminoma Reveals the Cellular and Molecular Characteristics of Metastatic Cell Lineage

**DOI:** 10.3389/fonc.2022.871489

**Published:** 2022-04-12

**Authors:** Linjian Mo, Zhenyuan Yu, Yufang Lv, Jiwen Cheng, Haibiao Yan, Wenhao Lu, Cheng Su, Qiang Ling, Zengnan Mo

**Affiliations:** ^1^ Center for Genomic and Personalized Medicine, Guangxi Key Laboratory for Genomic and Personalized Medicine, Guangxi Collaborative Innovation Center for Genomic and Personalized Medicine, Guangxi Medical University, Nanning, China; ^2^ Department of Urology, The First Affiliated Hospital of Guangxi Medical University, Nanning, China

**Keywords:** testicular seminoma, single-cell RNA sequencing, scRNA-seq, lymph node metastasis, tumor-immune microenvironment

## Abstract

**Background:**

Testicular cancer is the most common solid malignancy in young men. Given the many histological classifications of testicular tumors, seminoma is one of the most treatable cancers. The survival rate in early-stage disease was more than 90%. Thus, seminoma at the cellular and molecular levels, especially at the single-cell level, is worth studying.

**Methods:**

We performed a single-cell RNA sequencing (scRNA-seq) study on a patient who was diagnosed with testicular seminoma with lymph node metastasis. This study presented tumor tissue, PBMC, pelvic and renal hilus lymph node in a total of 18,206 high-quality single-cell transcriptome information. The characteristics of metastatic cell lineage were revealed by the comparison between different tumor cell subtypes at the scRNA level.

**Results:**

A single-cell map of testicular seminoma with lymph node metastasis was constructed by scRNA-seq. We discovered the gene expression characteristics of the tumor cells in testicular seminoma, especially metastatic tumor cells. *KRT8* and *KRT18* were commonly expressed in the three tumor cell subtypes. However, *TCF7L1*, *SCG3* and *SV2C* were the specifically expressed genes of tumor cell subtypes in primary tumor sites. Some molecular markers specifically expressed by the metastatic cell lineage, such as *POU5F1*, were identified.

**Conclusions:**

We revealed the molecular characteristics of testicular seminoma at the single-cell level, especially the metastatic tumor cells. This study could provide new insights into the diagnosis and treatment of testicular seminoma.

## Introduction

Testicular cancer is the most common solid malignancy in young men, and its incidence has increased worldwide over the past two decades (1–3). According to the WHO classification system, testicular cancer was defined into germ cell neoplasia *in situ-*related germ cell tumors and non-germ cell neoplasia *in situ-*related germ cell tumors (4). Testicular germ cell tumors (TGCT) are histologically subdivided into seminoma and nonseminoma (4). Most testicular cancers are germ cell tumors, and half of these are seminomas (5). Seminoma accounts for about a third of all testicular germ cell malignancies and is one of the most treatable cancers (6). Although 90% of patients with seminoma had a good prognosis, lymph node metastasis often occurs (5).

Previous studies indicated that the pathogenesis of testicular seminoma may be associated with anomalies in the short arm of chromosome 12 (7, 8). Another view is that the natural formation of the spermatogonial niche is disrupted, resulting in germ cell death and subfertility or infertility and ultimately testicular seminoma (9). Some somatic mutations in the genes encoding *KIT*, GTPase KRAS and cell division cycle protein 27 homologue (*CDC27*) may promote the development of testicular seminoma, and some treatment-refractory cases of this disease showed mutations in the gene encoding the DNA repair protein *XRCC2* (10–12). Somatic mutations appear to facilitate progression when invasive lesions occur, and these mutations may contribute to the genetic heterogeneity of testicular seminoma (3). Therefore, the gene expression characteristics of testicular seminoma may be associated with its development, progress and metastasis. However, single-cell transcriptome studies of testicular seminoma have never been reported. Single-cell RNA sequencing (scRNA-seq) can reveal the cell characteristics at single-cell level.

High-throughput scRNA-seq (13) technology has been developed and applied to many urinary cancers, such as bladder cancer, prostate cancer and renal cell carcinoma (14–17). In this study, we performed scRNA-seq for testicular seminoma that presented pelvic and renal hilus lymph node metastases. With scRNA-seq, the single-cell transcriptome characteristics of testicular seminoma were identified, especially the lineage of metastatic tumor cells.

## Materials and Methods

### Ethics Approval and Participation Consent

This study was approved by the Institutional Review Board (IRB) of The First Affiliated Hospital Guangxi Medical University, and the patient signed the informed consent.

### Testicular Seminoma Sample

**Figure 1:**
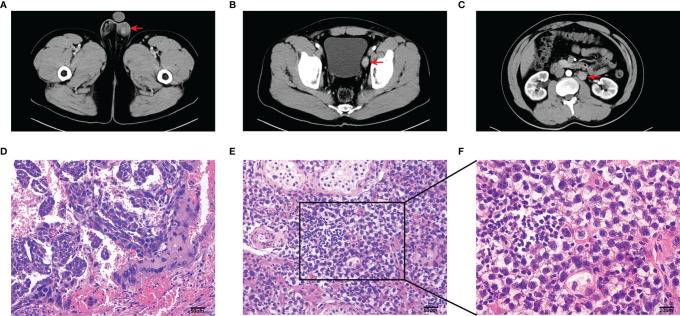
Imaging and pathological information of this sample. **(A–C)** Computed tomography (CT) plain and arterial images of primary testicular tumor **(A)**, pelvic lymph node **(B)** and renal hilus lymph node **(C)** metastases. Tumor and lymph node metastases (red arrow). **(D–F)** Photomicrographs of HE stained sections in the sample of testicular tumor. Scale bar, 50 um **(D, E)**. Scale bar, 20 um **(F)**.

The sample was obtained from a 27-year-old male patient undergoing testicular tumor resection and laparoscopic pelvic and renal hilus lymph node dissection in the First Affiliated Hospital Guangxi Medical University in China. Preoperative CT examination of the patient indicated testicular tumor with pelvic lymph node (LN1) and renal hilus lymph node (LN2) metastases (**Figures 1A**–**C**). Postoperative pathological results confirmed that testicular seminoma (**Figures 1D**–**F**) was complicated with lymph node metastasis. The patient had a past medical history of cryptorchidism and underwent testicular descent exopexy.

### Preparation of Testicular Seminoma Single-Cell Suspension

Samples of testicular seminoma were transferred into a 50 ml tube with transfer buffer (HBSS, Gibco, C11875500BT; 3% FBS, Gibco, 10099141 and 1% penicillin/streptomycin, Gibco, 15140122). The samples were transported in ice boxes for 30 min.

The tissue of testicular seminoma was transferred into a dish on ice, and cold transfer buffer was added to cover the tissue. After washing the tissue thrice with cold DPBS, it was cut into small pieces using sterile scalpel. The pieces were transferred into a 15 ml tube. Pieces were washed twice with 10 ml of DPBS (centrifuge in 350 x g for 5 min), and the supernatant was aspirated using a vacuum pump. The volume of tissue pellet was estimated (typically ~1 ml), and 14 ml pre-warmed digestion containing 1 mg/ml collagenase IV (Gibco, 17104019) and 1 mg/ml DNaseI (Roche, 10104159001) in HBSS were added. The mixture was incubated for 7 min at 37°C on a rocker at 300 rpm, and then, the digestion was stopped by adding RPMI1640 with 10% FBS. The tissue was resuspended by pipetting up and down, and the suspension was transferred into a new 15 ml of Falcon tube kept on ice. The remnant tissue was digested with 5 ml of TrypLE Express (Gibco, 12604039). The tissue was incubated at 37°C for 5 min, and this process was repeated twice for up to 15 min. The digestion was stopped by adding RPMI1640 with 10% FBS. The cell suspension was centrifuged for 5 min at 300 x g and at 4°C. The supernatant was aspirated using a vacuum pump. RBCs were removed by using 5 mL of 1X RBC lysis buffer (BioLegend, 420301) for 5 min on ice. The cell pellet was washed twice with 10 ml of DPBS (centrifuge in 350 x g for 5 min), and single cells were obtained by filtering through strainers with 100 µm mesh size (BD Falcon, 352360) and 40 µm (BD Falcon, 352340). Cells were resuspended in an appropriate volume of DPBS with 1% FBS to obtain testicular seminoma single-cell suspension. Cells were counted after 0.4% trypan blue solution (Gibco, 15250061) staining.

### Preparation of Lymph Node Single-Cell Suspension

The transfer buffer was prepared (3% FBS and 1% penicillin/streptomycin in HBSS) and placed on ice. We transported lymph node metastasis specimens with the transfer buffer to the laboratory within 30 min. Samples were transferred into a dish on ice, and cold transfer buffer was added to cover them. We carefully separated the lymph nodes (LNs) using sterile scissors and microforceps. LNs were light brown. After washing LN twice with DPBS, LNs were cut into 2 mm pieces with a sterile scissor. We prepared the digestion mixture (collagenase IV 1 mg/ml and DNaseI 1 mg/mL in HBSS) and placed it in a water bath at 37°C. Disrupted LN was transferred into a 15 ml tube, and 10 ml of the digestion mixture was added. Then, the mixture was incubated for 25 min at 37°C and with slow shaking during digestion. After 25 min, digestion was stopped by the addition of RPMI1640 medium containing 10% FBS. The digestion solution was poured onto a 100 µm cell strainer to filter out large tissue fragments. Cells were washed twice with pre-cold DPBS and centrifuged at 300 x g for 5 min at 4°C. The pellet was incubated in 5 ml of RBC lysis buffer for 5 min on ice to deplete RBCs and filtered through a 40 μm cell strainer to obtain a single-cell suspension. Single-cell was washed twice with cold DPBS and centrifuged at 300 x g for 5 min at 4°C. Cells were resuspended in DPBS (containing 1% FBS). We counted viable cells under a light microscope after staining them with trypan blue.

### Preparation of PBMC Single-Cell Suspension

Approximately 3 ml of whole blood from the patient was collected with his consent by a trained phlebotomist. We normally use EDTA and heparin, which are compatible with this method. The blood was diluted at a ratio of 1:1 with HBSS in sterile 15 ml tubes. The diluted blood was pipetted slowly on top of lymphocyte separation medium (Solarbio, P8610) in 15 ml centrifuge tubes, and the volumes of lymphocyte separation medium and diluted blood were 1:2. Centrifugation was conducted for 20 min at 500 x g (accelerator 1, brake 1, and 4°C). We discarded the upper layer (plasma) and obtained the whitish buffy coat (where the PBMCs are) that laid above the Ficoll histopaque layer, thereby avoiding the red cell lower layer. PBMCs were washed twice with 10 ml of DPBS (WISENT, 311-425-CL) and centrifuged in 350 x g for 5 min. RBCs were removed using 5 mL of 1x RBC lysis buffer (BioLegend, 420301) for 5 min on ice. Then, they were filtered through a 40 µm cell strainer (BD Falcon, 352340). PBMCs were washed twice with 10 ml of DPBS (centrifuge in 350 x g for 5 min) and resuspended with DPBS.

### Single-Cell cDNA Library Construction and Sequencing

We performed scRNA-seq for the single-cell suspensions according to the standard protocol in the user’s Guide for *10X Genomics Chromium Single Cell 3’ Reagent Kit V3* (https://support.10xgenomics.com/single-cell-gene-expression/index/doc/user-guide-chromium-single-cell-3-reagent-kits-user-guide-v3-chemistry). The sequencing files (.bcl) were converted to FASTQ files by CellRanger (version 3.1.0), and FASTQ files were compared with the human genome reference sequence GRCh38. The detailed process was presented in our previous studies (16, 17). Finally, we obtained the files to process the downstream analysis, including a barcode table, a feature table and a gene expression matrix.

### Seurat for Quality Control (QC) and Secondary Analysis

R (version 4.0.2, https://www.r-project.org/) and Seurat (18, 19) R package (version 4.0.0, https://satijalab.org/seurat/) were used for secondary analysis. In the beginning, we performed the QC of the data. In general, one cell should contain at least 500 genes, and the number of genes was less than twice of the median number of detected genes (potential cell duplets). The proportion of mitochondrial genes was recommended to be less than 10% (**Figures S1A, B**). According to the above conditions, we filtered the cells obtained from the primary analysis and finally obtained high-quality cells for downstream analysis (**Table 1**).

**Table 1:** Details for each sample (The “Range of genes” and “Range of Mitochondrial genes” were the parameters of quality control).

Sample	Cell number	Median genes	Median reads	Range of genes	Range of mitochondrial genes	Cell number after QC
tumor	6,204	2,716	50,930	500∼5,000	<10%	4,568
LN1	8,888	1,438	37,566	500∼3,000	<10%	5,694
LN2	5,643	1,674	49,049	500∼3,000	<10%	4,487
PBMC	6,156	1,670	55,645	500∼3,000	<10%	3,457

First, we use the *Merge* function in the Seurat package to merge all samples (tumor, LN1, LN2 and PBMC), and then use the Harmony (20) package to eliminate batch effect and generate UMAP. Here, we chose 20 principal components (PCs) and a resolution of 0.5 as the appropriate cell clustering parameter.

After normalisation, the highly variable genes were found by calculating the relationship between average expression and dispersion. We used the top 2000 variable genes for downstream analysis. Cell cycle analysis was performed based on a previous study (21). Using the *Jackstraw* function, we calculated the significant PCs from each sample. We chose 20 PCs as the appropriate cell clustering parameter that affected the distribution of uniform manifold approximation and projection (UMAP). At a resolution of 0.5, all cells were clustered by the *FindClusters* function from Seurat. We then used *FindAllMarkers* to calculate differentially expressed genes (DEGs) between each cell type (**Tables S1**–**S4**).

### Reconstructing Cell Differentiation Trajectories by RNA Velocity and Monocle2

Firstly, we used RNA velocity (http://velocyto.org, version 0.6) (22) to estimate the direction and rate of differentiation of Leydig cells. Using the Bam file generated by Cellranger processing, we apply the *velocyto* function to generate the.loom file. Subsequently, the SeuratWrappers package made it easy to integrate Seurat objects with.loom files. Finally, the rate and direction of each cell were calculated using *RunVelocity* function. Here, we set deltaT to 1, kCells to 25, and fit.quantile to 0.02.

The Monocle2 (23) R package (version 2.10.1) was used to reconstruct the cell fate decisions and pseudo-time trajectories of Leydig cells in testicular tumor. After the Leydig cell subtypes were classified by Seurat, the differentiation trajectory of different cell subtypes was reconstructed. We used the genes that were expressed in at least 10 cells and in greater than 5% of cells. For this point, the analysis code was in *Figshare* (https://figshare.com/s/41c8d392f0f3450a66e7). On the other hand, the key genes that influenced by the trajectory were identified after using “orderCells” and “differentialGeneTest” function in Monocle2. Then, the parameters (thresholds on the cell local density and the nearest distance) were used to determine the number of clusters. The top 1000 most significant DEGs from each Leydig cell subtypes were used for the set of ordering genes and dimension reduction. Finally, we reconstructed a trajectory and discovered the key genes that influenced the trajectory.

### Copy Number Variation (CNV) Analysis of Single-Cell Data

The R package inferCNV (https://github.com/broadinstitute/inferCNV) was used to perform CNV analysis on this scRNA-seq data. By applying this R package, the chromosome copy number characteristics of each cell can be estimated. In this study, we estimated CNV from tumor cell types, and used NK cells as the control group. For 10x Genomics single-cell data, the cut-off value was 0.1.

### Comparison of Different Samples

We calculated the overall similarity of gene expression between the different samples by evaluating the average expression level of each gene in different samples. We obtained the average expression levels of the same gene from different samples and then conducted Pearson’s correlation coefficient for all genes in different samples. The average gene expression of each gene was transformed by the formula *log1p*. Using the same method, Pearson’s correlation coefficient for the same cell type between two different samples was also compared.

### Gene Ontology (GO) Enrichment Analysis on Tumor Cells

GO enrichment analysis was performed on tumor cell types. The top 50 DEGs (**Tables S1**, **S2**) from tumor sample (cluster 9) and LN1 (cluster 11) were selected for GO enrichment analysis (24) (http://geneontology.org/). The biological processes of tumor cells were enriched and analysed, and 15 of the most significant biological processes were revealed. However, tumor cell 2 (cluster 17 in tumor sample) did not undergo enrichment on significant biological processes.

### Ligand-Receptor Interactions of Each Cell Types in LN1

The interaction among cells was analysed by using cellphoneDB (25) (https://www.cellphonedb.org/). CellPhoneDB is a publicly available repository that includes receptors, ligands and their interactions (25). We first analysed all types of cells that were clustering LN1 from Seurat. We then process the data into a matrix format that cellphoneDB can directly evaluate.

## Results

### A Single-Cell Map of Testicular Seminoma With Lymph Node Metastasis Was Constructed by scRNA-Seq

**Figure 2:**
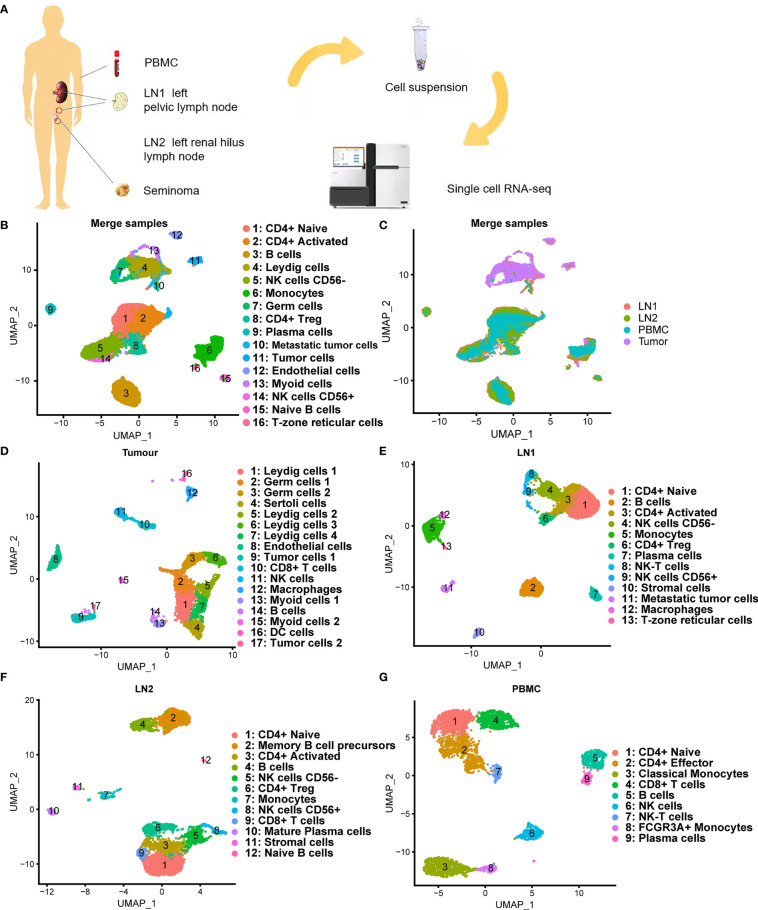
Single-cell transcriptomic atlas of testicular seminoma sample. **(A)** Schematic design of the overall design. **(B, C)** Single-cell transcriptomic map of testicular tumor (Tumor), left pelvic lymph node (LN1), left renal hilus lymph node (LN2) and peripheral blood mononuclear cells (PBMC), with 16 distinct cell types. **(D)** UMAP plot representation of testicular seminoma (Tumor) with 17 distinct cell types. **(E)** UMAP plot representation of left pelvic lymph node (LN1) with 13 distinct cell types. **(F)** UMAP plot representation of left renal hilus lymph node (LN2) with 12 distinct cell types. **(G)** UMAP plot representation of PBMCs with 9 distinct cell types.

scRNA-seq was performed on tumor tissue, lymph node tissues (including 2 lymph nodes with tumor cell metastasis) and PBMC of a patient who was diagnosed with testicular seminoma with lymph node metastasis (**Figure 2A**). We prepared single-cell suspensions of tissue samples by enzyme digestion (*Materials and Methods*). Then, the prevalent 10X Genomics instrument was used for scRNA-seq (**Figure 2A**). After QC by Seurat (18, 19), 4,568, 5,694, 4,487 and 3,457 high-quality single-cell transcriptome data were captured from tumor tissue, pelvic lymph node (LN1), renal hilus lymph node (LN2) and PBMC, respectively (**Table 1**). Cluster analysis of a total of 18,206 single-cells after merging showed that lymph nodes and PBMC had very similar cell types, whereas tumor tissue had relatively specific cell types (**Figures 2B, C**). Interestingly, the distribution of metastatic tumor cells provided by LN1 were closer to that of tumor tissue (**Figures 2B, C**). Subsequently, we performed cluster analysis on each tissue sample separately and found that tumor tissue can be classified into 17 different cell types (clusters 1, 2, 3, 4, 5, 6, 7, 8, 9, 10, 11, 12, 13, 14, 15, 16 and 17), as follows: Leydig cells 1, germ cells 1, germ cells 2, Sertoli cells, Leydig cells 2, Leydig cells 3, Leydig cells 4, endothelial cells, tumor cells 1, CD8+ T cells, NK cells, macrophages, Myoid cells 1, B cells, Myoid cells 2, DC cells and tumor cells 2, respectively (**Figure 2D**). Cluster analyses of pelvic lymph node and renal hilus lymph node were very similar with 13 and 12 cell types, respectively (**Figures 2E, F**). Interestingly, we discovered tumor cells, which we named metastatic tumor cells, in the pelvic lymph node sample (**Figure 2E**). PBMC can be classified into nine different cell types, namely, CD4+ Naïve, CD4+ Effector, classical monocytes, CD8+ T cells, B cells, NK cells, NK-T cells, FCGR3A+ monocytes and plasma cells (**Figure 2G**). Non-immune cells were more abundant in testicular seminoma tissue, whereas lymph nodes and PBMC were more abundant in immune cells (**Figures S1C**–**F**).

**Figure 3:**
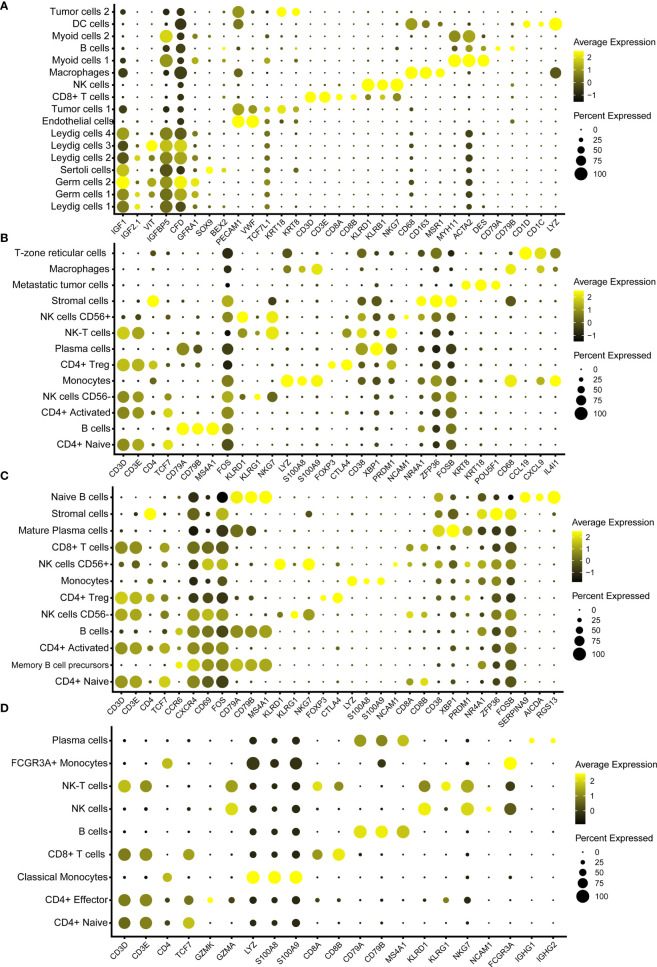
Bubble chart showing the marker genes of each cluster in testicular tumor **(A)**, left pelvic lymph node **(B)**, left renal hilus lymph node **(C)** and PBMCs **(D)**.

We used bubble maps to show the marker genes for each cell type and defined the cell types by these marker genes (**Figures 3A**–**D**). Obvious differences were found in the gene expression in each cell type. These marker genes are derived from previous studies (3, 26–31). For example, tumor cells showed particularly high expressions of *KRT8*, *KRT18* and *TCF7L1*, whereas endothelial cells showed particularly high expressions of *PECAM1* and *VWF*. (**Figure 3A**).

### scRNA-Seq Discovered the Diversity of Leydig Cells

**Figure 4:**
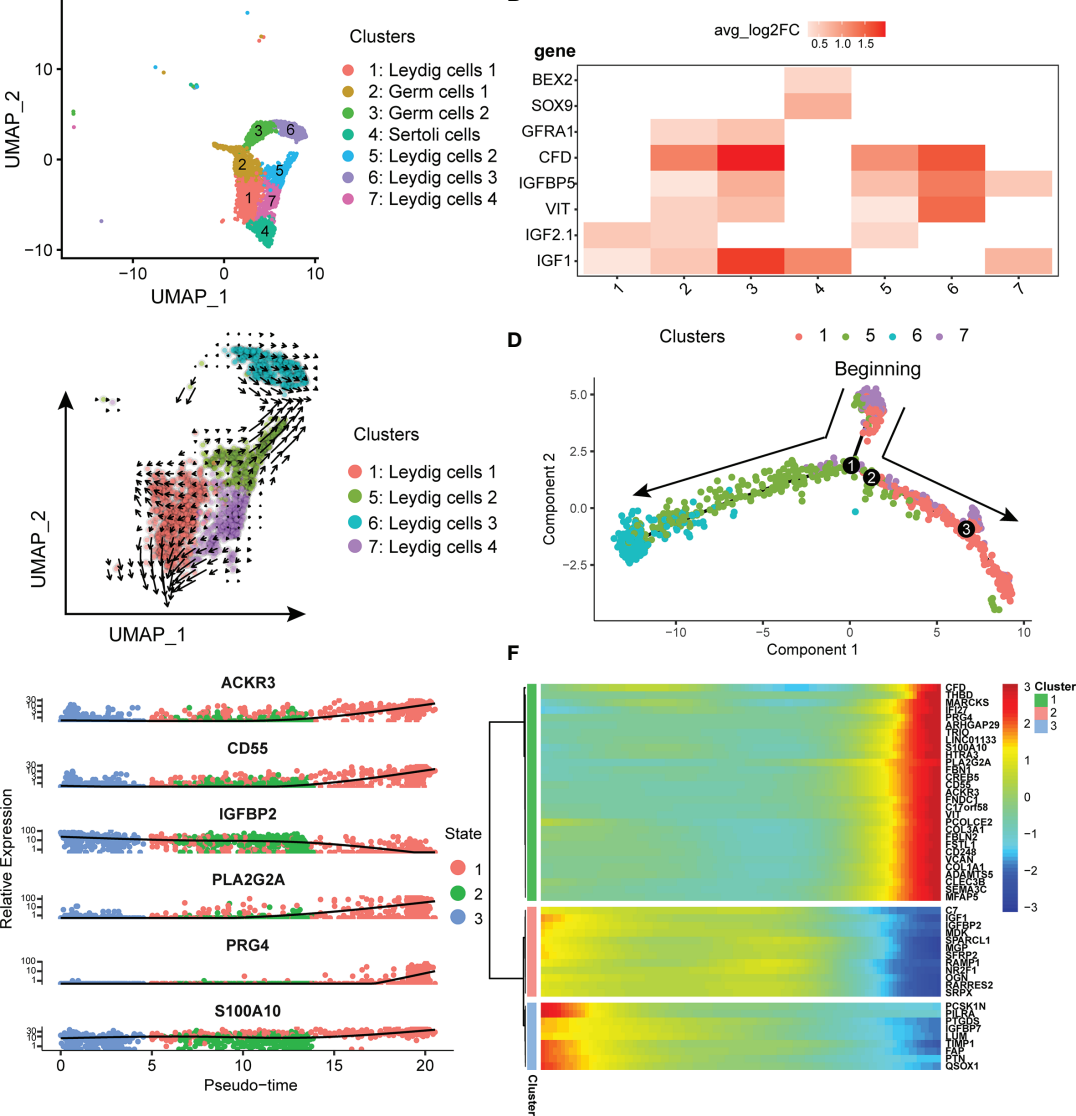
**(A)** UMAP plot of germ, Leydig and Sertoli cells in testicular tumor. **(B)** Markers of Leydig, germ and Sertoli cells in testicular tumor; avg_log2FC, average log2 fold change. **(C)** Pseudotime analysis of Leydig cells by using RNA velocity reconstructed the developmental lineages (arrows represent average velocity). **(D)** Reconstruction of the cell fate decisions and pseudo-time trajectories of Leydig cells in testicular tumor by Monocle2. **(E)** Scatter plots showing the expression levels and changes in relative expression of key genes that affected the evolution stage. **(F)** Heatmap showing the top 50 genes that influenced the trajectory.

In previous single-cell studies of normal testis, Leydig cells were of a single type (26). However, our results discovered that Leydig cells in testicular seminoma tissue can be classified into four subtypes (**Figure 4A**). In addition to Leydig cells, we identified some normal testicular cell types, such as germ and Sertoli cells, in testicular seminoma. Considering that *GFRA1* is the marker gene of germ cells, and *SOX9* and *BEX2* are the marker genes of Sertoli cells, we distinguished these two cell types from Leydig cells (**Figure 4B**). Leydig cells expressed *IGF1*, *IGF2*, *VIT*, *IGFBP5* and *CFD* (**Figure 4B**).

To confirm the initial state of Leydig cells, RNA velocity (22) was used to estimate the direction and rate of Leydig cell differentiation. RNA velocity can be used for analysis of developmental lineages and cellular dynamics in scRNA-seq (22). According to the results of RNA velocity, we discovered that Leydig Cells 4 was in the initial state (**Figure 4C**). Next, monocle (23) was used to reconstruct the development trajectory of Leydig cells. Leydig cell 4 was the cell type in the initiation stage, and Leydig cell 1 and 2 was the cell type in the transition stage, whereas Leydig cells 3 was the cell types in the terminal stage (**Figure 4D**). Based on the trajectory analysis, the most significant five genes affecting the development trajectory were *ACKR3*, *CD55*, *IGFBP2*, *PLA2G2A*, *S100A10* and *PRG4* (**Figure 4E**). The expressions of these genes decreased with further cell differentiation. Some genes affected cell differentiation, and we selected the top 50 most significant genes for clustering display (**Figure 4F**). We have classified these genes into three different clusters according to the initial, transitional and terminal stages of development trajectory (**Figure 4F**).

### scRNA-Seq Revealed the Gene Expression Characteristics of the Tumor Cells in Testicular Seminoma, Especially Metastatic Tumor Cells

**Figure 5:**
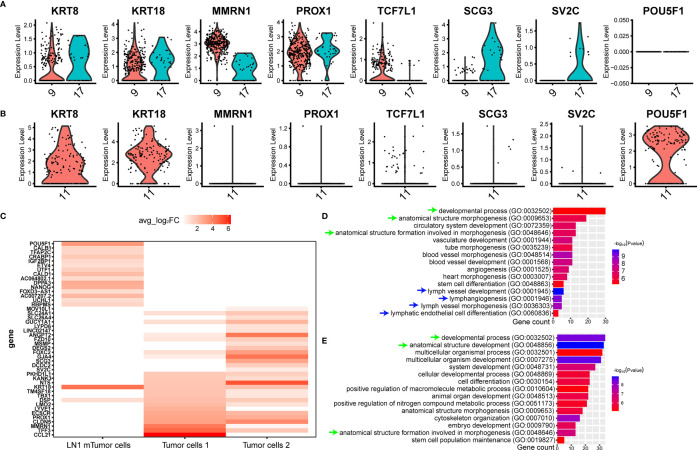
ScRNA-seq revealed the cellular molecular characteristics of testicular seminoma cells. **(A)** Violin plots showing tumor-specific markers expressed in tumor cell types from testicular tumor. **(B)** Violin plots showing tumor-specific markers expressed in tumor cells from left pelvic lymph node tumor. **(C)** Gene expression characteristics of the tumor cells in primary testicular tumor and pelvic lymph node metastasis. **(D)** GO enrichment analysis of tumor cells 1 in primary testicular tumor. **(E)** GO enrichment analysis of tumor cells in pelvic lymph node metastasis for biological process.

We isolated and captured two tumor cell subtypes from tumor tissue, such as tumor cells 1 (cluster 9) and tumor cells 2 (cluster 17), by scRNA-seq (**Figure 2D**). In addition, both cell types specifically expressed *KRT8*, *KRT18*, *MMRN1* and *PROX1* (**Figure 5A**). However, some significant differences in gene expression were found between the two tumor cell subtypes. *TCF7L1*, which is a marker gene for testicular seminoma in a previous study (30), was specifically expressed in tumor cells 1, whereas *SCG3* and *SV2C* were specifically expressed in tumor cells 2 (**Figure 5A**). Interestingly, metastatic tumor cells were captured in pelvic lymph nodes (LN1), and the gene expression characteristics were revealed by scRNA-seq. Compared with tumor cells at the primary site, metastatic tumor cells still retained high expressions of *KRT8* and *KRT18*, whereas metastatic tumor cells no longer expressed *MMRN1*, *PROX1*, *TCF7L1*, *SCG3* and *SV2C* (**Figure 5B**). Metastatic tumor cells expressed another marker gene for seminoma, *POU5F1* (**Figure 5B**). *POU5F1*, also named *OCT4*, has been identified as a marker gene for seminoma previously (3). In addition, metastatic tumor cells also specifically expressed some genes, such as *DPPA3*, *NANOG*, *CRABP1* and others (**Figure 5C**). We discovered some markers in the three different tumor cell subtypes by scRNA-seq (**Figure 5C**).

### scRNA-Seq Revealed the Variations in Cellular and Molecular Characteristics of Testicular Seminoma During Metastasis

We initially performed GO analysis on both tumor cell types at primary site and tumor cell type at LN1 metastasis. (*Materials and Methods*). However, only tumor cell 1 and metastatic tumor cell were able to enrich significant biological processes, while tumor cell 2 was not found. Many similar biological processes can be found between tumor cells 1 and metastatic tumor cells, such as ‘developmental process’, ‘anatomical structure morphogenesis’ and ‘anatomical structure formation involved in morphogenesis’ (**Figures 5D, E**). Interestingly, we found that tumor cells 1 were also active in some lymphoid related biological processes, such as ‘lymph vessel development’, ‘lymphangiogenesis’, ‘lymph vessel morphogenesis’ and ‘lymphatic endothelial cell differentiation’ (**Figure 5D**).

**Figure 6:**
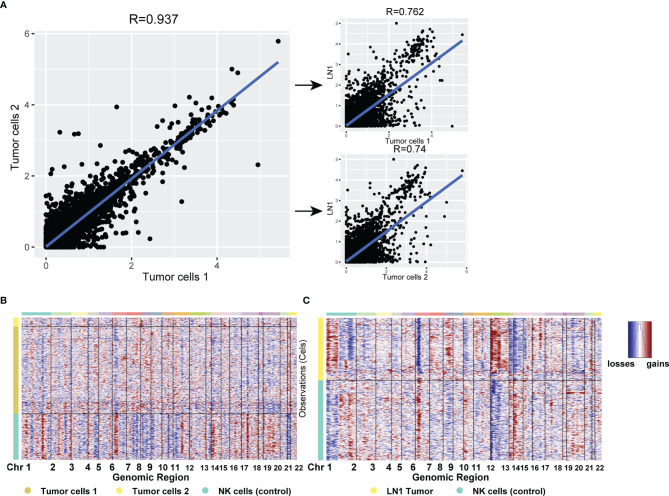
**(A)** Scatterplot showing the log1p of the average expression (AE) per gene in different tumor cell types from primary testicular tumor and pelvic lymph node metastasis. **(B, C)** CNV analysis for all cell types in testicular tumor **(B)** and pelvic lymph node metastasis **(C)**. Copy number gains (red) and losses (blue).

Considering that the DEGs of tumor cells 2 could not be used to enrich the biological process from GO database, we further analysed the expression of each gene from the three tumor cell subtypes. Firstly, we compared the expression of each gene between tumor cells 1 and tumor cells 2 in tumor samples. The similarity of gene expression between tumor cells 1 and tumor cells 2 was very high, and the Pearson’s correlation coefficient was 0.937 (**Figure 6A**). This finding indicated that different subtypes of tumor cells still retained good homology and stability in the primary tumor site. Subsequently, we compared tumor cells 1, tumor cells 2 and metastatic tumor cells and noted their similarities. The similarity of gene expression between tumor cells 1 and metastatic tumor cells was superior to that of tumor cells 2, as shown by Pearson’s correlation coefficient (0.762 VS 0.74). Considering that the biological process was associated with lymph nodes, as found by GO analysis, metastatic tumor cells from tumor cells 1 were more likely to be metastatic. Thus, we hypothesised that the tumor cells 1 could be the cell type that metastasised to pelvic lymph nodes. However, our results showed that the similarity of tumor cells was reduced after metastasis from the primary site to pelvic lymph nodes (**Figure 6A**). CNV analysis was performed on three different tumor subtypes. Considering the stability of CNV in immune cells, we used NK cells as a control group. CNV analysis showed that the copy number variation of tumor cells 1 and tumor cells 2 was similar (**Figure 6B**). However, metastatic tumor cells featured a gained copy number of chromosome 12 (**Figure 6C**). The genetic marker of testicular tumor was the anomalies of the short arm of chromosome 12 (7, 8). Thus, the gained copy number of chromosome 12 was also likely to be a characteristic of metastatic tumor cells.

### The Similarity Between Immune Cells of Lymph Node Infiltrated by Tumor and Peripheral Blood Immune Cells at Single-Cell Level

**Figure 7:**
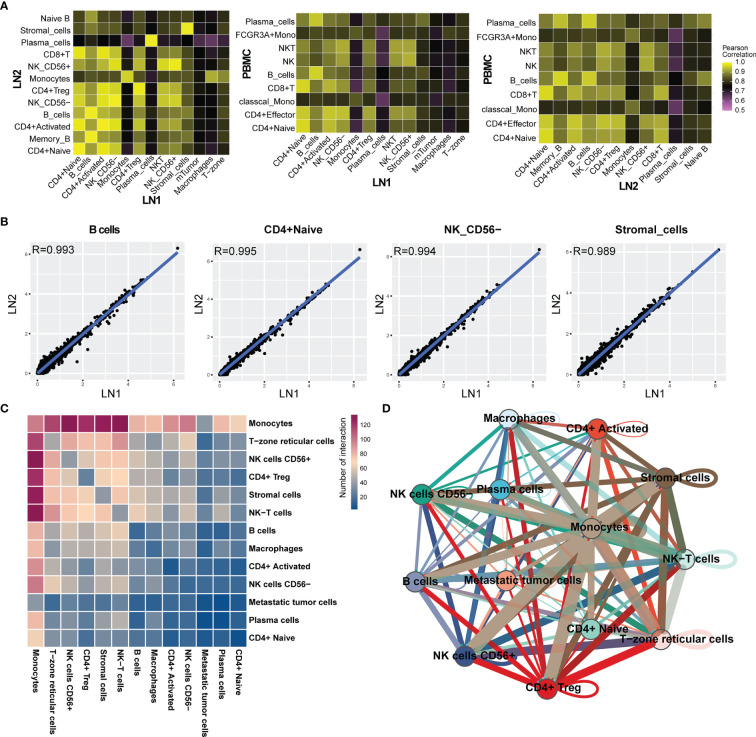
**(A)** Heat map indicating Pearson correlations on the averaged profiles among each cell types for left pelvic lymph node (LN1) and left renal hilus lymph node (LN2) and PBMC. **(B)** Scatterplot showing the log1p of the average expression per gene of B cells, CD4+ naive T cells, CD56- NK cells and stromal cells in LN1. **(C)** Ligand–receptor interactions across each cell type in LN1; the colour represents the number of interactions. **(D)** Analysis of cell-cell communication of each cell type by Cell chat. Nodes represent the cluster, and the line thickness represents number of interactions.

In this study, LN1 was infiltrated by tumor cells, whereas LN2 and PBMC were not. Firstly, after comparing each cell type in LN1 and LN2, the gene expression was very similar between the same cell types, as shown by with Pearson correlation coefficients greater than 0.9 (**Figure 7A**). The same result also appeared in the comparison of LN1 or LN2 with PBMC (**Figure 7A**). In the comparison of LN1 and LN2 cell types, we discovered the highest similarity between B cells, CD4+ Naive, NK cells CD56- and stromal cells, which was close to 1 (**Figure 7B**). Thus, this suggested that the gene expression of immune cells in lymph nodes was not significantly altered even in the presence of tumor cell infiltration.

Subsequently, we analysed the association between all cell types in LN1 through ligand–receptor interactions. Monocytes had the highest number of ligand-receptor interactions with all immune cells and were closely associated with almost all cell types except metastatic tumor cells (**Figures 7C, D**). However, the interactions between metastatic tumor cells and the immune cells were minimal (**Figures 7C, D**). We hypothesised that the immune microenvironment was inadequate in the interaction between metastatic tumor cells. This may be a biological feature of testicular seminoma.

## Discussion

As one of the neoplasms of the genitourinary system, testicular tumors are often neglected due to their lower incidence than prostate cancer, bladder cancer and renal cell carcinoma (1). Considering the variety of testicular tumors, the prognosis of different histological types of testicular tumors varies (4). Therefore, an in-depth study of testicular tumors at the cellular level helps further reveal the causes of their occurrence or progression. To date, no scRNA-seq study has been performed on testicular tumors. By scRNA-seq, we revealed the characteristics of tumor cells at the single-cell level of a case of diagnosed testicular seminoma with pelvic lymph node metastasis.

Our results showed that there were four Leydig cell subtypes in testicular seminoma (**Figure 4A**). In a previous study, the researchers found only one subtype of Leydig cells in normal testicular tissue (26). Considering that the samples in this study were tumor samples from cryptorchidism, the cell composition of the testis was different from that of normal testis. Therefore, our results showed the cellular composition characteristics of immature testis.

scRNA-seq is a powerful technique especially when used for profiling gene expression in a small number of cells. In this study, although the number of tumor cells was small, results revealed their molecular characteristics well. By comparing the primary site tumor cells with lymph node metastases, we identified the genes specifically expressed in metastatic tumor cells (**Figure 5A**). The gene expression of metastatic tumor cells has rarely been reported previously. This is also an important goal of this study. Information on the genetic characteristics of metastatic tumor cells may provide a reference for the treatment and diagnosis of seminoma in the future. In addition, we discovered that gene expression in tumor cell 1 was more similar to that in metastatic tumor cells than that in tumor cell 2 (**Figure 6A**). GO analysis revealed some common biological processes in both cell types (**Figure 5D**). Thus, based on the results of scRNA-seq, we can infer which cell subtype was more prone to metastasis in the primary site. Considering that the samples for scRNA-seq came from only one patient, this study had some limitations. Although postoperative pathology reported tumor cell metastasis in renal hilus lymph nodes, our results did not capture tumor cells in LN2; due to different samples, the single-cell suspension is not the same as the pathological results of the sample. Fortunately, the cell transcriptome information captured in each of our samples was of high quality.

We revealed the molecular characteristics of testicular seminoma at the single-cell level, especially the metastatic tumor cells. This study could provide new insights into the diagnosis and treatment of testicular seminoma.

## Data Availability Statement

The code for analysing single-cell data and raw data after processing can be accessed in Figshare (https://doi.org/10.6084/m9.figshare.18502661). The raw data and processed data in this manuscript could be accessed in the NCBI GEO Datasets (GSE197778, https://www.ncbi.nlm.nih.gov/geo/query/acc.cgi?acc=GSE197778).

## Ethics Statement

The studies involving human participants were reviewed and approved by the Institutional Review Board (IRB) of The First Affiliated Hospital Guangxi Medical University. The patients/participants provided their written informed consent to participate in this study. Written informed consent was obtained from the individual(s) for the publication of any potentially identifiable images or data included in this article.

## Author Contributions

LM performed the scRNA-seq experiments and wrote the paper. ZY performed scRNA-seq analyses, created the figures, and wrote the paper. YL performed the scRNA-seq experiments and wrote the paper. JC, HY, CS, WL, and QL provided and dissected testicular seminoma tissues. YL and WL provided assistance during establishing library. ZM conceived and supervised the project, analysed the data, created the figures, and wrote the paper. All the authors read the manuscript and made comments.

## Funding

This work was supported by the grants from the National Natural Science Foundation of China (81770759), the National Key R&D Program of China (2017YFC0908000), Major Project of Guangxi Innovation Driven (AA18118016), Guangxi Key Laboratory for Genomic and Personalised Medicine [grant number 16-380-54, 17-259-45, 19-050-22, 19-185-33, 20-065-33].

## Conflict of Interest

The authors declare that the research was conducted in the absence of any commercial or financial relationships that could be construed as a potential conflict of interest.

## Publisher’s Note

All claims expressed in this article are solely those of the authors and do not necessarily represent those of their affiliated organizations, or those of the publisher, the editors and the reviewers. Any product that may be evaluated in this article, or claim that may be made by its manufacturer, is not guaranteed or endorsed by the publisher.

## Supplementary Material

Supplementary Figure 1
**(A)** Quality control of the scRNA-seq data. nFeature, number of genes; nCount and unique molecular identifiers; and percent.mt and percentage of mitochondrial genes. **(B)** Relationship between the percentage of mitochondrial genes and the mRNA reads and between the amount and reads of mRNA. **(C–F)** Proportion of each cell type in testicular tumor **(C)**, left pelvic lymph node **(D)**, left renal hilus lymph node **(E)** and PBMCs **(F)**.
