# Supplementary figures and images for: Single-Cell RNA Sequencing of Metastatic Testicular Seminoma Reveals the Cellular and Molecular Characteristics of Metastatic Cell Lineage
Source: Front Oncol. 2022 Apr 12;12:871489. doi: 10.3389/fonc.2022.871489 (PMC9039315; doi:10.3389/fonc.2022.871489)

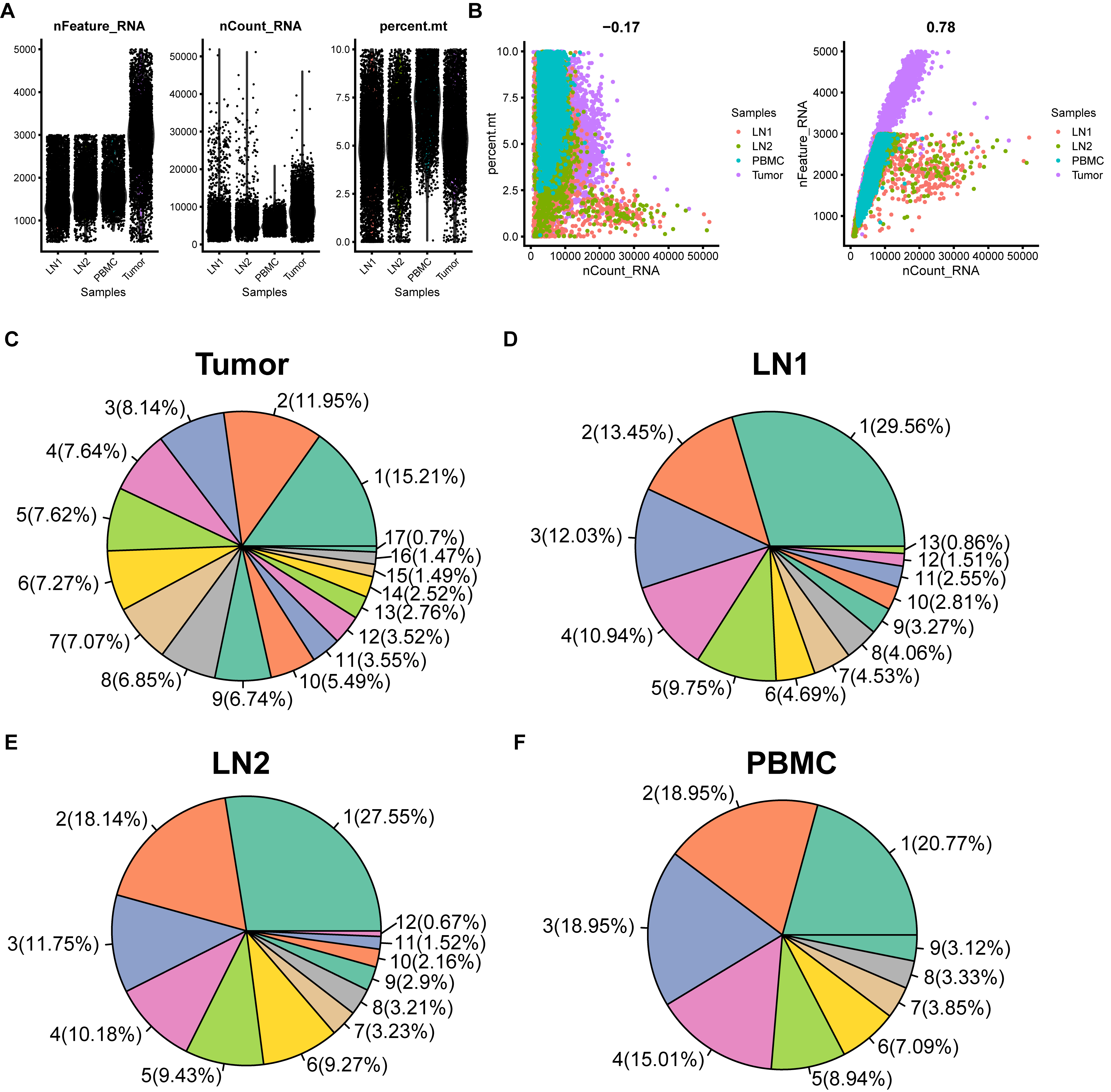

Supplement: Supplementary Figure 1 — (A) Quality control of the scRNA-seq data. nFeature, number of genes; nCount and unique molecular identifiers; and percent.mt and percentage of mitochondrial genes. (B) Relationship between the percentage of mitochondrial genes and the mRNA reads and between the amount and reads of mRNA. (C–F) Proportion of each cell type in testicular tumor (C), left pelvic lymph node (D), left renal hilus lymph node (E) and PBMCs (F). [file Image_1.tif]
